# Hippocampus volume and plasma‐derived extracellular vesicle TDP‐43 levels as biomarkers for LATE‐NC

**DOI:** 10.1002/alz.086373

**Published:** 2025-01-09

**Authors:** Michel J. Grothe, Jesús Silva‐Rodríguez, Diana Ortega‐Cruz, Francisco J. López‐González, Minerva Martinez‐Castillo, Selçuk Oezdemir, Alberto Rabano, Pascual Sanchez‐Juan, Anja Schneider

**Affiliations:** ^1^ Reina Sofia Alzheimer Center, CIEN Foundation, ISCIII, Madrid, Madrid Spain; ^2^ Reina Sofia Alzheimer Center, CIEN Foundation, ISCIII, Madrid Spain; ^3^ Centre for Biomedical Technology, Universidad Politécnica de Madrid, Madrid, Madrid Spain; ^4^ German Center for Neurodegenerative Diseases (DZNE), Bonn Germany; ^5^ Reina Sofia Alzheimer Centre, CIEN Foundation, ISCIII, Madrid Spain

## Abstract

**Background:**

Limbic‐predominant age‐related TDP‐43 encephalopathy neuropathologic change (LATE‐NC) is a common neuropathologic finding at advanced age that associates with hippocampal sclerosis (HS) and is often comorbid with AD pathology. Neuroimaging measurements of LATE‐NC‐associated limbic degeneration have been proposed as indirect biomarkers, but molecular‐specific biomarkers for LATE‐NC are still lacking. Here we used combined ante‐mortem blood and MRI data to study TDP‐43 levels in plasma‐derived small extracellular vesicles (sEV‐TDP‐43) and hippocampal volume (HV) in relation to LATE‐NC and HS at autopsy.

**Method:**

We studied 56 autopsied dementia patients from the Reina Sofia Alzheimer Center (Madrid) who had ante‐mortem blood samples and quality‐controlled 3T‐MRI available. Plasma sEVs were isolated by size exclusion chromatography and TDP‐43 was quantified by SIMOA. HV was derived from structural MRI using Freesurfer. Neuropathological examination included assessment of AD neuropathologic change (ADNC), LATE‐NC, and presence of HS. Differences in plasma sEV‐TDP‐43 levels and HV between individuals with (+) and without (‐) LATE‐NC were assessed using t‐tests and ROC curve analysis. Additional analyses stratified cases by presence/absence of HS and limited the sample to those with high ADNC.

**Result:**

Plasma sEV‐TDP‐43 levels were significantly higher (p=0.02) and HV (p=0.03) was significantly lower in LATE‐NC(+)(n=35) vs LATE‐NC(‐)(n=21), although group separation in ROC analysis was relatively weak (AUC_sEV‐TDP‐43_ = 0.65; AUC_HV_ = 0.68). Combining both biomarkers increased group separation to AUC_sEV‐TDP‐43+HV_ = 0.76. HV was highly sensitive to HS status (p=0.001), whereas sEV‐TDP‐43 was not (p=0.45). As a consequence, sEV‐TDP‐43 also distinguished between LATE‐NC(‐) and LATE‐NC(+) without HS (p=0.02, AUC=0.72), whereas HV did not (p=0.17). When limited to cases with high ADNC, sEV‐TDP‐43 distinguished between those with and without comorbid LATE‐NC (p=0.03, AUC=0.64), but HV did not (p=0.37).

**Conclusion:**

Plasma sEV‐TDP‐43 levels and hippocampal volume provide complementary information as biomarkers of LATE‐NC. While hippocampal volume is a sensitive biomarker of LATE‐NC‐associated neurodegeneration, plasma sEV‐TDP‐43 levels appear to be more directly linked to the pathology. The relatively low group separation could be partly due to the characteristics of the study sample focused on end‐of‐life dementia patients with late stage disease and multiple comorbidities.

